# Assessment of *Erythrobacter* Species Diversity through Pan-Genome Analysis with Newly Isolated *Erythrobacter* sp. 3-20A1M

**DOI:** 10.4014/jmb.2012.12054

**Published:** 2021-02-03

**Authors:** Sang-Hyeok Cho, Yujin Jeong, Eunju Lee, So-Ra Ko, Chi-Yong Ahn, Hee-Mock Oh, Byung-Kwan Cho, Suhyung Cho

**Affiliations:** 1 Department of Biological Sciences, Korea Advanced Institute of Science and Technology, Daejeon 34141, Republic of Korea; 2 KI for the BioCentury, Korea Advanced Institute of Science and Technology, Daejeon 34141, Republic of Korea; 3 Biological Resource Center, Korea Research Institute of Bioscience and Biotechnology, Daejeon 34141, Republic of Korea

**Keywords:** *Erythrobacter*, whole-genome sequencing, pan-genome analysis, secondary metabolites

## Abstract

*Erythrobacter* species are extensively studied marine bacteria that produce various carotenoids. Due to their photoheterotrophic ability, it has been suggested that they play a crucial role in marine ecosystems. It is essential to identify the genome sequence and the genes of the species to predict their role in the marine ecosystem. In this study, we report the complete genome sequence of the marine bacterium *Erythrobacter* sp. 3-20A1M. The genome size was 3.1 Mbp and its GC content was 64.8%. In total, 2998 genetic features were annotated, of which 2882 were annotated as functional coding genes. Using the genetic information of *Erythrobacter* sp. 3-20A1M, we performed pangenome analysis with other *Erythrobacter* species. This revealed highly conserved secondary metabolite biosynthesis-related COG functions across *Erythrobacter* species. Through subsequent secondary metabolite biosynthetic gene cluster prediction and KEGG analysis, the carotenoid biosynthetic pathway was proven conserved in all *Erythrobacter* species, except for the spheroidene and spirilloxanthin pathways, which are only found in photosynthetic *Erythrobacter* species. The presence of virulence genes, especially the plant-algae cell wall degrading genes, revealed that *Erythrobacter* sp. 3-20A1M is a potential marine plant-algae scavenger.

## Introduction

Among marine microorganisms, *Erythrobacter* species contribute to carbon distribution in the ocean [1]. While diverse microorganisms participate in the carbon cycle via autotrophic inorganic carbon fixation or heterotrophic organic carbon redistribution, photoheterotrophic *Erythrobacter* species potentially mediate both organic and inorganic carbon cycles [2-4]. Photoheterotrophic bacteria called aerobic anoxygenic phototrophic bacteria (AAPB), which include *Erythrobacter* species, such as *E. litoralis* and *E. longus*, harvest light energy using photosynthetic gene clusters (PGCs) [5]. The non-AAPB strains were discovered after the establishment of the *Erythrobacter* genus, in which photosynthetic marine bacteria were classified [6]. The photosynthetic ability of *Erythrobacter* species is shared with purple phototrophic bacteria, which are phylogenetically closely related [7].

*Erythrobacter*, namely the red (erythro-) bacteria (-bacter), are well-known carotenoid-producing microorganisms [8]. Carotenoids are a family of bioactive, yellow to orange colored, pigment compounds used medicinally for their antioxidant properties, disease risk-reducing benefits, and enhancement of immune functions [9]. Carotenoid derivatives are produced from terpenoid backbone precursors, such as geranyl-geranyl pyrophosphate, which is also produced from pyruvate and glyceraldehyde 3-phosphate via the terpene biosynthetic pathway. *E. longus*, for example, produces various carotenoids, including β-carotene, zeaxanthin, nostoxanthin, caloxanthin, β-cryptoxanthin, rubixanthin, bacteriorubixanthin, anhydrorhodovibrin, and erythroxanthin [8]. *Erythrobacter* species have different carotenoid production profiles depending on their genetic composition. In particular, AAPB species produce unique carotenoids named spheroidene or spirilloxanthin, which are utilized as photosynthetic pigments [7, 10].

Owing to the development of next-generation sequencing techniques and subsequent bioinformatics tools, it is now possible to obtain massive amounts of genetic information. Based on genetic information, the phylogeny of new taxa can be identified more precisely. In addition, metabolite profiles can be predicted based on reconstructed metabolic pathways, and the ecological niche can be studied based on genetic profiles [11, 12]. In this study, we sequenced the genome of *Erythrobacter* sp. 3-20A1M. We report the complete genome sequence of this species and its genetic characteristics through gene annotations. Additionally, the distinguishable characteristics of *Erythrobacter* sp. 3-20A1M were identified by comparing its secondary metabolite profile with that of other *Erythrobacter* species through pan-genome analysis.

## Materials and Methods

### Isolation and Scanning Electron Microscopy

*Erythrobacter* sp. 3-20A1M was isolated from the southern sea of GeoJe, South Korea (34°46′ N, 128°46′ E), in August 2016 and deposited in the Korea Collection for Type Cultures (KCTC; Accession #, KCTC 18715P). The specimen was cultured in marine broth 2216 (BD Difco, USA) or marine agar at 25°C. For scanning electron microscopy (SEM), *Erythrobacter* sp. 3-20A1M was cultured in marine broth and centrifuged at 4,000 rpm at 4°C. The bacterial cell pellet was resuspended in a 2.5% paraformaldehyde-glutaraldehyde mixture buffered with 0.1 M phosphate (pH 7.2). The sample was fixed in the solution for 2 hours, post-fixed in 1% osmium tetroxide in the same buffer for 1 hour, dehydrated in graded ethanol, which was then substituted with isoamyl acetate. They were then dried at a critical point in CO_2_. Finally, the samples were sputtered with gold in a sputter coater (SC502, Polaron) and observed using a scanning electron microscope and FEI Quanta 250 FEG (FEI, USA).

### Genome Sequencing and Genome de novo Assembly

Genomic DNA of *Erythrobacter* sp. 3-20A1M was extracted using the Wizard Genomic DNA Purification Kit (Promega) following the manufacturer’s protocol. The quality of the extracted genomic DNA was assessed by a NanoDrop TM2000 (Thermo Fisher Scientific, USA) for a UV absorbance ratio (260:280) of ~2 and inspection using 1% agarose gel electrophoresis. A genome sequencing library with an insert size of 550 bp was prepared using the TruSeq Nano DNA Library Prep Kit (Illumina, USA) following the manufacturer’s protocol. The prepared genome sequencing library was sequenced using a 250-cycle paired-end reaction on the Illumina MiSeq platform. Raw sequencing reads were subjected to PlasmidSeeker to detect native plasmids [13]. Sequencing data were processed using CLC Genomics Workbench 6.5.1. software (CLC Bio, Denmark). PhiX, adapters, and quality trimmed reads were used for de novo genome assembly (word size = 24, bubble size = automatic, and mapping option = map reads back to contigs (slow)). The assembled genome sequence was assessed using Benchmarking Universal Single-Copy Orthologs (BUSCO). The complete genome sequence was deposited in GenBank under accession number CP045200.

### Phylogenetic Analysis

Genomic sequences of each strain used for phylogenetic analysis were downloaded from the NCBI Genome Portal (Table S1). The phylogenetic tree was reconstructed using the Up-to-date Bacterial Core Gene (UBCG) analysis pipeline [14]. Randomized Axelerated Maximum Likelihood (RAxML) was used to generate the phylogenetic tree from the calculated distance data [15]. 16s rRNA based phylogeny analysis was conducted using MEGA-X [16]. The evolutionary history was inferred using the Neighbor-Joining method and the evolutionary distances were computed using the p-distance method.

### Gene Annotation and Secondary Metabolite Biosynthesis Gene Prediction

The *de novo* assembled genome sequence of *Erythrobacter* sp. 3-20A1M was annotated using the NCBI Prokaryotic Genome Annotation Pipeline [17]. Subsequently, the amino acid sequences were extracted from the annotated coding genes and searched against KEGG Orthology (KO) ID, Clusters of Orthologous Groups (COG), and Gene Ontology (GO) using EggNOG-mapper (version 2) [18-21].

### Pan-Genome Analysis and Prediction of Secondary Metabolite Clusters

For the pan-genome analysis of *Erythrobacter* species, the pan-genome analysis pipeline (PGAP) (version 1.2.1) was used [22]. Ortholog clusters were organized using the coding sequences (CDSs) of each genome with the gene family (GF) method under default parameters (E-value: 1e10, score: 40; identity: 50; coverage: 50). To generate additional COG input data, all *Erythrobacter* genomes used were functionally annotated with EggNOG-Mapper (version 2) with their amino acid sequences. Secondary metabolite biosynthetic gene clusters were predicted using antiSMASH (version 5.2.0) [23]. The assembled genome sequence was assessed using BUSCO [24].

## Results and Discussion

### Phenotypic and Genotypic Identification of *Erythrobacter* sp. 3-20A1M

**Fig. 1:**
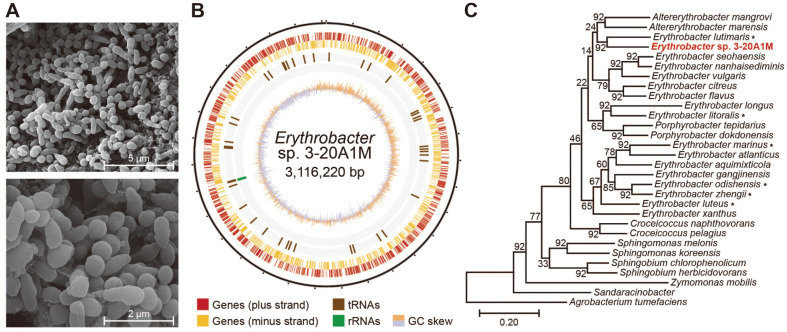
Identification of *Erythrobacter* sp. 3-20A1M. **A**. Scanning electron micrograph of *Erythrobacter* sp. 3-20A1M. **B**. Circular representation of the *Erythrobacter* sp. 3-20A1M complete genome. From the outside to the center: genome (black, ticks every 100 Kbp), genes on the plus strand (red), genes on the minus strand (yellow), tRNA (brown), rRNA (green), and the GC skew (orange and light purple). **C**. Phylogenetic analysis of *Erythrobacter* sp. 3-20A1M and 29 closely related taxa, performed based on their core genes. *Agrobacterium tumefaciens* was selected as the outgroup. The tree is drawn to scale, with branch length units equivalent to those of evolutionary distances used to infer the phylogenetic tree. The evolutionary distances were provided by Up-to-date Bacterial Core Gene (UBCG) and plotted by Randomized Axelerated Maximum Likelihood (RAxML).

First, the morphological features of *Erythrobacter* sp. 3-20A1M were observed. As the name suggests, *Erythrobacter* is a bacterium with a yellow to orange color, and the cell pellet of *Erythrobacter* sp. 3-20A1M also had an orange color. The cell shape of the bacteria was observed using SEM (Fig. 1A). The bacteria had an irregular cell shape under the SEM. The shape ranged from spherical to rod-shaped. The length and breadth of the cell was 0.5–2.0 μm and 0.5 μm, respectively. It has been reported that *Erythrobacter* species use binary fission for reproduction; however, irregular divisions, such as budding or Y cell division, are also seen at higher growth rates [10]. In addition, a glue-like substance was identified on the cell surface in SEM images, which confirmed that *Erythrobacter* sp. 3-20A1M forms a biofilm.

We performed genome sequencing and genome assembly to analyze the genetic component of *Erythrobacter* sp. 3-20A1M. The genome assembly provided a complete genome of 3.1 Mbp (Fig. 1B). The quality of the assembled genome was assessed using BUSCO, and duplicated or fragmented orthologs were not found (Table S2) [24]. The DNA GC content of the genome was 64.8%. The assembled genome sequence was annotated using the NCBI Prokaryotic Genome Annotation Pipeline [17]. As a result, 2998 features were annotated (Table 1 and Supplementary Dataset S1). Of the 2946 annotated CDSs, 2830 functional CDSs were identified, excluding 116 pseudogenes. The remaining 52 features were RNA genes, including genes of three complete rRNAs (5S, 16S, and 23S), 45 tRNAs, and four non-coding RNAs.

To identify and confirm the evolutionary relationship of the newly sequenced bacterium, we performed a phylogenetic analysis based on core *Erythrobacter* sp. 3-20A1M genes with 16 other *Erythrobacter* species and 12 other species from the order Sphingomonadales, which are closely related to *Erythrobacter* (Fig. 1C and Table S1). *Agrobacterium tumefaciens* was included as an outgroup for the analysis of 30 species. The evolutionary distance was calculated using UBCG and the phylogenetic tree was visualized using RAxML [14, 15]. *Erythrobacter*, within the order Sphingomonadales and the family Sphinogomonadaceae, were grouped under branches in close proximity, and *Erythrobacter* sp. 3-20A1M formed a single phylogenetic cluster with *E. lutimaris*. In addition, Porphyrobacter within the same order and family as *Erythrobacter*, was close to *Erythrobacter* species in phylogenetic distance. However, Sphingomonas, classified in the same order and family as *Erythrobacter*, and Croceicoccus, classified in the order Sphingomonadales and the family *Erythrobacteraceae*, were far from *Erythrobacter* species in phylogenetic distance [25, 26]. The 16s rRNA sequence-based phylogeny analysis also supports the novelty of the newly identified *Erythrobacter* specimen (Fig. S1).

### Functional Categorization

**Fig. 2:**
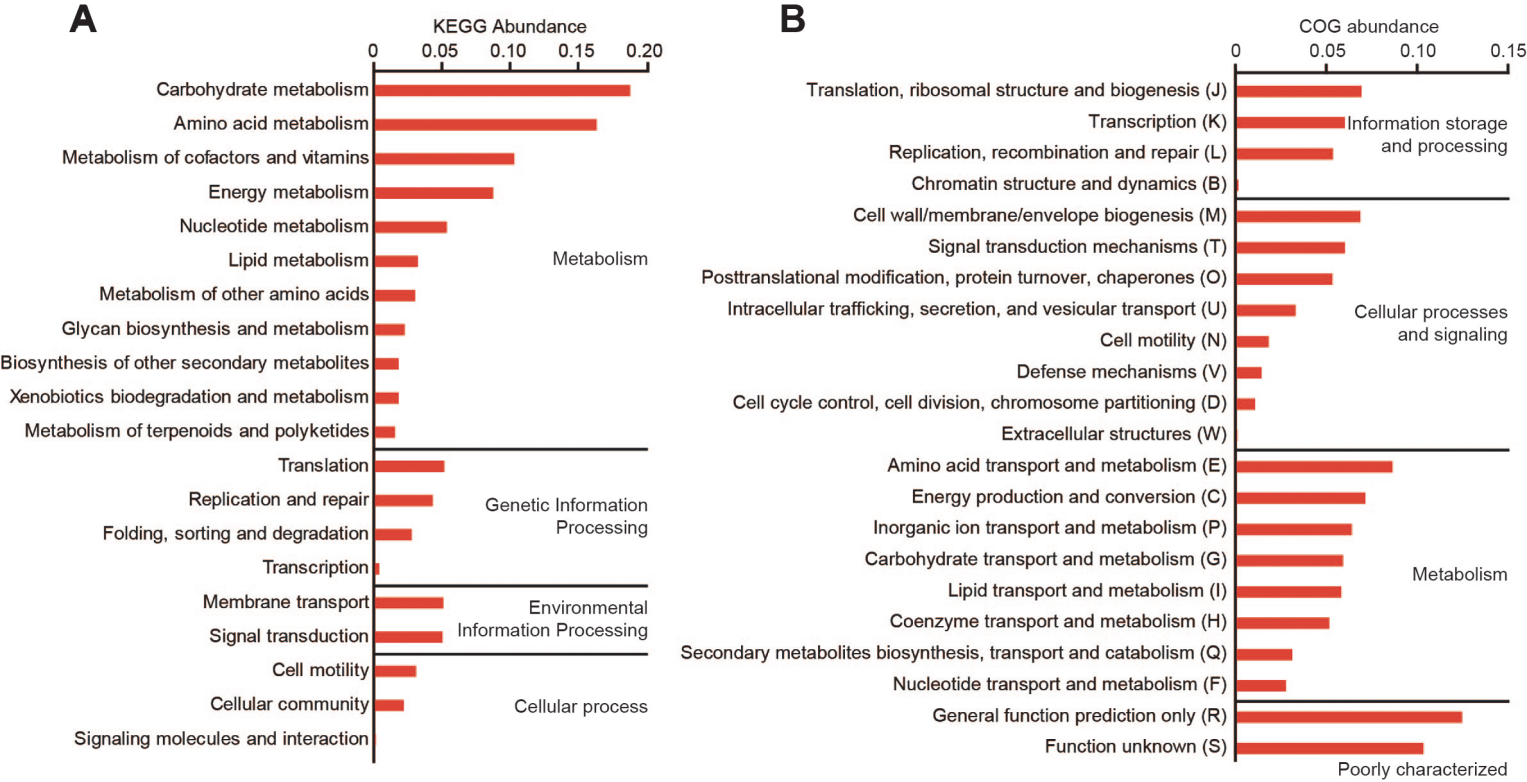
KEGG pathway analysis and COG analysis. EggNOG-mapper analysis of 2882 *Erythrobacter* sp. 3-20A1M coding genes. **A**. KEGG Orthology categorized 1731 genes. **B**. Clusters of Orthologous Groups categorized 2481 genes.

The functional composition of the encoded genetic features in *Erythrobacter* sp. 3-20A1M was categorized according to KO, COG, and GO [18-21]. A total of 2882 coding genes were assigned to 1731 KO IDs, 2481 COG functions, and 685 GO terms (Fig. 2 and Table S3). In the KEGG analysis, genes involved in carbohydrate and amino acid metabolism were abundant, similar to most microorganisms. In particular, genes related to the metabolism of cofactors and vitamins were ranked high, reflecting the higher carotenoid synthesis level of *Erythrobacter* producing its red-orange color (Fig. 2A). Moreover, genes related to membrane transport and signal transduction, involved in environmental information processing, were present. This suggests that *Erythrobacter* sp. 3-20A1M may play a role in the exchange of cellular metabolites with other microorganisms. In COG analysis, the genes for amino acid transport and metabolism (F); energy production and conversion (C); and translation, ribosomal structure, and biogenesis (J) were abundant, except for poorly categorized categories (R and S)(Fig. 2B). It also has a high percentage of genes involved in inorganic ion transport and metabolism.

### Pan-Genome Analysis of 17 *Erythrobacter* Species

**Fig. 3:**
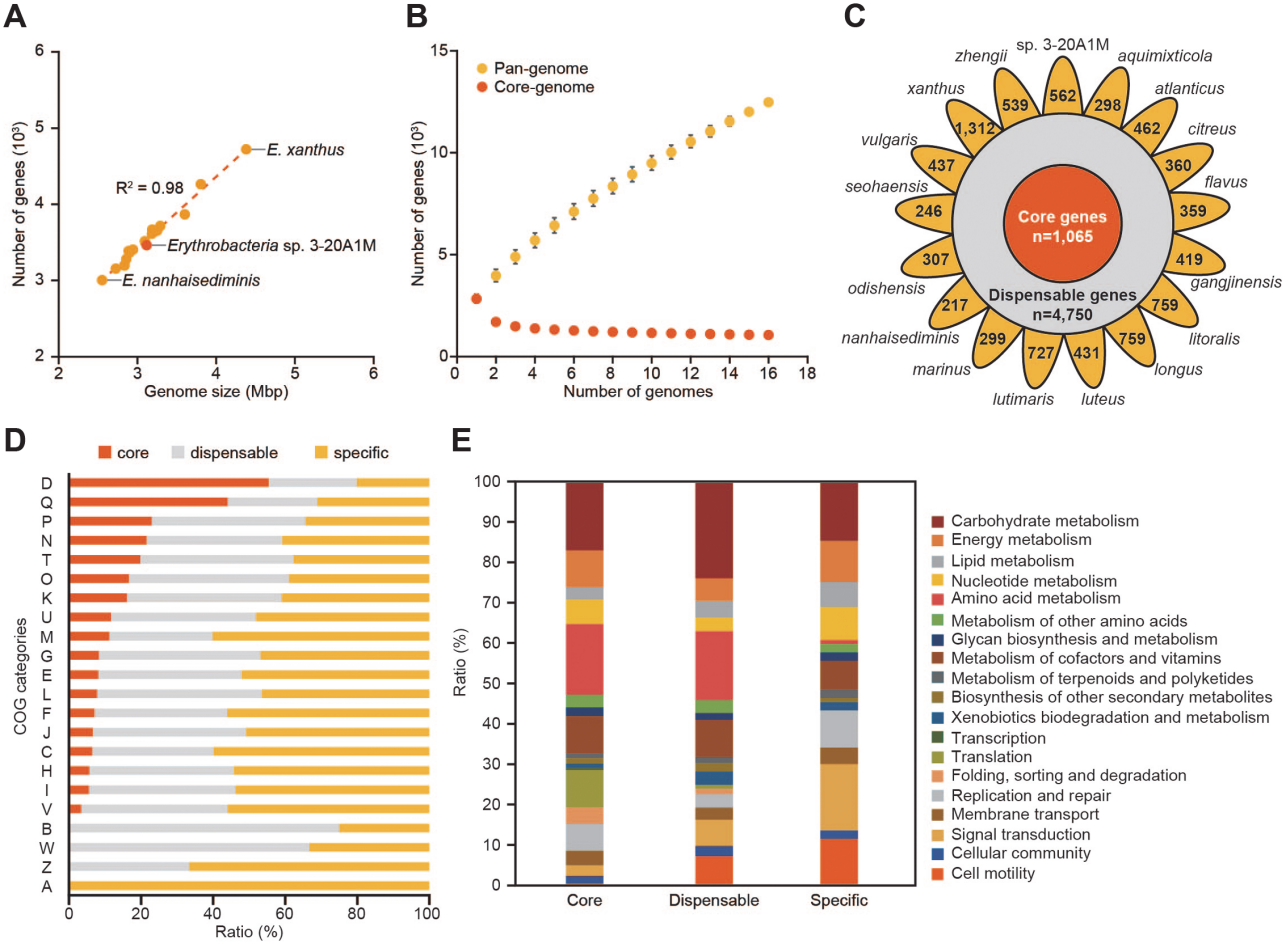
Pan-genome analysis of *Erythrobacter* species. **A**. Correlation between the genome size and the number of coding genes of each genome. **B**. Pan-genome and core-genome profiles. The number of genes in the *Erythrobacter* pangenome and core-genome are plotted. **C**. Pan-Genome analysis pipeline was used to analyze 17 *Erythrobacter* species. The number of common core genes across all 17 species is presented in the innermost circle (red). The number of dispensible genes is presented in the middle circle (gray). The number of genes specific to each strain is presented in the outmost area (yellow). **D**. The distribution of core, dispensible, and strain-specific genes presented as a bar graph. COG categories: D, Cell cycle control, cell division, and chromosome partitioning; Q, Secondary metabolites biosynthesis, transport, and catabolism; P, Inorganic ion transport and metabolism; N, Cell motility; T, Signal transduction mechanisms; O, Post-translational modification, protein turnover, and chaperones; K, Transcription; U, Intracellular trafficking, secretion, and vesicular transport; M, Cell wall/ membrane/envelope biogenesis; G, Carbohydrate transport and metabolism; E, Amino acid transport and metabolism; L, Replication, recombination and repair; F, Nucleotide transport and metabolism; J, Translation, ribosomal structure and biogenesis; C, Energy production and conversion; H, Coenzyme transport and metabolism; I, Lipid transport and metabolism; V, Defense mechanisms; B, Chromatin structure and dynamics; W, Extracellular structures; Z, Cytoskeleton; A, RNA processing and modification. **E**. The KEGG Orthology distribution among core, dispensible, and specific genes of *Erythrobacter* sp. 3-20A1M.

We conducted pan-genome analysis of 17 *Erythrobacter* species, including *Erythrobacter* sp. 3-20A1M (Table S1). Their genome sizes varied between a minimum of 2.6 Mbp in *E. nanhaisediminis* to a maximum of 4.4 Mbp in *E. xanthus* (Fig. 3A), while their GC content ranged from 57.4% to 67.2%.

In the PGAP, the pan-genome size of *Erythrobacter* species increased with an increasing number of species, implying that the pan-genome of *Erythrobacter* species belongs to the open pan-genome category (Fig. 3B) [22, 27]. A total of 1065 *Erythrobacter* genes were conserved across the 17 species, and thus were denoted as core genes (Fig. 3C). The core genes of *Erythrobacter* species accounted for 26.3% to 40.2% of all genes, and *Erythrobacter* sp. 3-20A1M had a core-genome ratio of 35.2%. At least two species maintained 4750 that were classified as dispensable genes. The number of unique genes in a species varied from 217 to 1312, with *E. xanthus* having the largest genome size and the largest number of unique genes.

We compared the COG category-based variability in the distributions of PGAP classifications, including core, dispensable, and specific (Fig. 3D). As a result, the core gene ratio was the highest with COG functions related to cell cycle control, cell division, and chromosome partitioning (D). Interestingly, the second-highest core gene proportion was found in the secondary metabolite biosynthesis, transport, and catabolism (Q) COG function. Since *Erythrobacter* species are known to produce carotenoids, it was inferred that the genes related to carotenoid biosynthesis might have been attributed to the high conservation ratio of the secondary metabolite related gene functions.

In the KEGG analysis, *Erythrobacter* sp. 3-20A1M showed noticeable ratios of dispensable and specific genes in the signal transduction and cell motility categories (Fig. 3E). In the signal transduction category, the specific genes belonged to a sub-category, the two-component system. Genes related to flagella assembly and bacterial chemotaxis, which are sub-categories of the cell motility category, are also included in the two-component system according to KEGG categorization (Table 2). *Erythrobacter* sp. 3-20A1M-specific genes belonging to flagella assembly included *flgKM*, *fliMS*, and *motB*, and bacterial chemotaxis included *cheABR*, *fliM*, and *motB*. Although other *Erythrobacter* species also have genes corresponding to the same KO ID, they were recognized as different gene families in the PGAP analysis. Among the two-component systems that did not correspond to the above two categories, genes related to virulence were included. In particular, *Erythrobacter* sp. 3-20A1M has three genes involved in exopolysaccharide biosynthesis, *epsAPC* (F7D01_05380, F7D01_13155, and F7D01_13520), of which epsAP was found to be a unique gene [28]. *Erythrobacter* sp. 3-20A1M also has the *pme* (pectinesterase, F7D01_10375) gene, encoding a plant cell wall-degrading enzyme, which was also found in the closest related species, *E. lutimaris*. Pectinesterase is an enzyme that decomposes pectin polysaccharides of plant cell walls. Regarding the marine ecosystem, pectin is also a component of algal cell walls [29]. These findings suggest a role of these two species as scavengers of pectin-containing organisms.

To further investigate the possible scavenging ability against plant-algae, the virulence factors of the *Erythrobacter* sp. 3-20A1M were searched using annotation data and KEGG categories. The virulence genes were searched under four categories: biofilm synthesis, secretion system, motility, and plant-algae cell wall degradation (Table S3). Additional to *epsAPC*, several exopolysaccharide biosynthetic genes and polysaccharide export genes were found [30]. *Erythrobacter* sp. 3-20A1M contains three intact bacterial secretion systems, which are the type IV secretion system, Sec-SRP system, and Tat system [31]. The strain contained an intact flagella machinery, which generates motility and also serves as a flagella type III secretion system [32]. By means of identifying the plant-algae cell wall degradation, the genes related to starch metabolism were searched. Along with the pectinesterase, glycosyl hydrolases, pectate lyase, cellulase, and cell wall hydrolase genes were identified that could be used as an arsenal for decomposing plant-algae cell walls.

### Secondary Metabolite Biosynthetic Gene Clusters of *Erythrobacter* Species

**Fig. 4:**
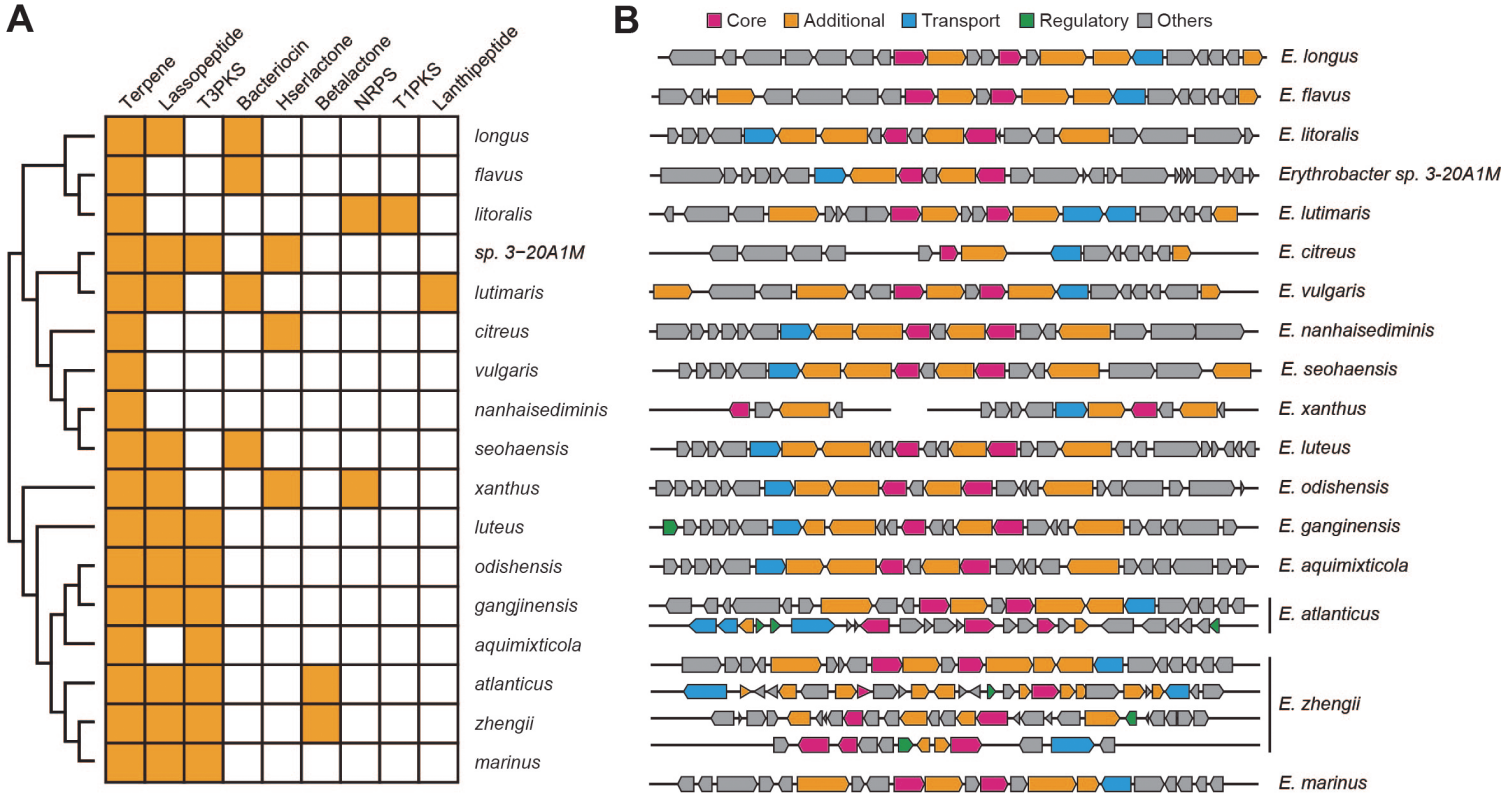
Secondary metabolites production of *Erythrobacter* species. **A**. Secondary metabolite biosynthetic gene clusters were predicted in 17 *Erythrobacter* species. Color indicates the presence of the biosynthetic gene cluster. The species were arranged by their phylogenetic relationships. **B**. Predicted terpene biosynthetic clusters are presented. Abbreviations: T3PKS, type 3 polyketide synthetase; Hserlactone, homoserine lactone; NRPS, non-ribosomal peptide synthase.

The secondary metabolite, including carotenoid, production capacity of the 17 *Erythrobacter* species was investigated through secondary metabolite biosynthetic gene cluster (BGC) prediction using the antiSMASH tool [23]. This analysis predicted 58 BGCs across the species (Table S4). Accordingly, the terpene BGC was predicted in all *Erythrobacter* species. In addition, the types of BGCs that are produced in each species were correlated with their phylogenetic relationships (Fig. 4A). In particular, the clade including *E. xanthus*, *E. luteus*, *E. odishensis*, *E. ganginensis*, *E. aquimixticola*, *E. atlanticus*, *E. zhengii*, and *E. marinus*, produces lasso peptide, type 3 polyketide synthetase (T3PKS), and the terpene biosynthetic cluster. Conversely, the lasso peptide and T3PKS were found less frequently in other clades. Interestingly, only *E. xanthus* was predicted to contain 13 BGCs. However, an error may have caused excessive predictions due to the relatively large number of contigs in the draft genome compared to that of other species, and the PGAP result also showed a large number of unique genes in *E. xanthus*. To assess the presence of erroneous genome sequences, the genome assembly quality was confirmed using BUSCO, returning a ratio of duplication of 0.7%. It was determined that the overall large number of genes or abundance of BGCs were not caused by duplication errors in the genome assembly process. While *Erythrobacter* sp. 3-20A1M was not phylogenetically close to the eight species mentioned above, it also produces lasso peptide, T3PKS, terpene, and has a BGC for homoserine lactone production. Among *Erythrobacter* species, the newly identified *Erythrobacter* sp. 3-20A1M produces diverse secondary metabolites.

The terpene biosynthetic pathway utilizes either the mevalonate (MVA) pathway or the methylerythritol 4-phosphate (MEP) pathway and starts with pyruvate and glyceraldehyde 3-phosphate generated in glycolysis [33]. Most organisms have only one of the pathways, and *Erythrobacter* species utilize the MEP pathway subsequent isoprenoid pathway to produce a terpenoid backbone precursor called geranyl-geranyl-pyrophosphate, which is used as a precursor of the carotenoid biosynthetic pathway. Next, we compared the terpene biosynthetic gene clusters of the 17 *Erythrobacter* species according to their functional roles (Fig. 4B). While all the terpene biosynthetic pathway genes were conserved among the 17 species based on KEGG analysis, the terpene BGCs predicted by antiSMASH were diversified. Most *Erythrobacter* species, including *Erythrobacter* sp. 3-20A1M, contain a single terpene BGC with two core genes, accessory proteins, and at least one transport gene, whereas *E. atlanticus* and *E. zhengii* have multiple terpene BGCs (Fig. 4B).

### Carotenoid Biosynthesis Pathway across *Erythrobacter* Species

**Fig. 5:**
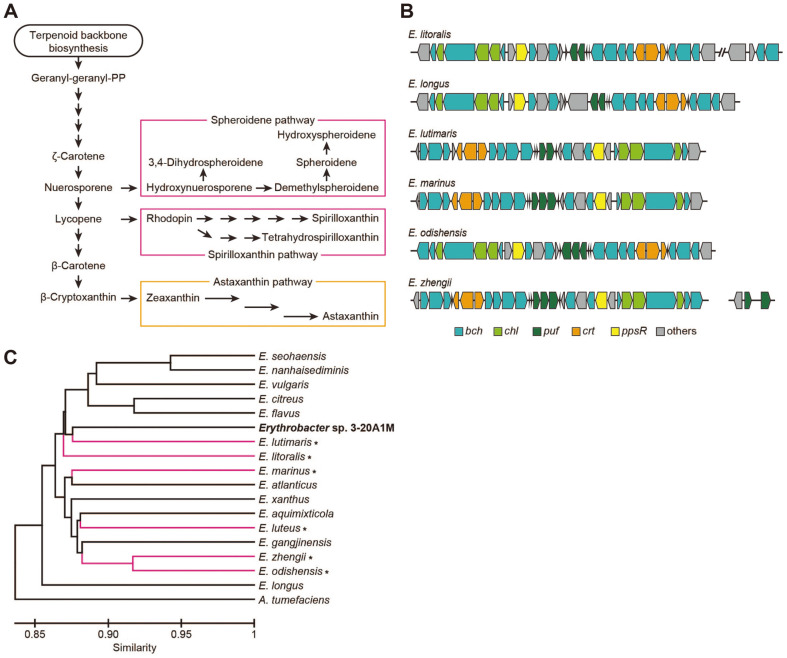
Carotenoid biosynthesis of *Erythrobacter* species. **A**. The carotenoid biosynthetic pathway is presented. The astaxanthin, spirilloxanthin, and spheroidene pathways are highlighted. **B**. Photosynthetic gene clusters are presented in 6 *Erythrobacter* species. **C**. Average nucleotide identity-based phylogeny tree of the 17 *Erythrobacter* species. Aerobic anoxygenic photosynthetic bacteria are highlighted with asterisks and indicated in magenta.

*Erythrobacter* species are known to produce excess carotenoids [8]. Carotenoid biosynthesis begins with geranyl-geranyl-pyrophosphate as a precursor generated from the terpene biosynthetic pathway (Fig. 5A). From the KEGG analysis, it was found that, except for *E. citreus*, all the *Erythrobacter* species studied have conserved pathways for zeaxanthin production from geranyl-geranyl-pyrophosphate with intermediate compounds, such as lycopene and β-carotene, and astaxanthin pathways to produce astaxanthin from zeaxanthin. However, six *Erythrobacter* species, *E. lutimaris*, *E. litoralis*, *E. marinus*, *E. luteus*, *E. zhengii*, and *E. odishensis*, have additional carotenoid biosynthesis pathways, including the spheroidene pathway and the spirilloxanthin pathway. These two pathways are used for pigment production in photosynthetic bacteria, such as purple bacteria. *Erythrobacter* species are phylogenetically close to purple sulfur bacteria, and this is the suspected reason for the presence of photosynthetic bacteria among *Erythrobacter* species [5, 10]. They are classified according to their photosynthetic ability, and the photosynthetic species are called AAPB. The difference in photosynthetic ability among *Erythrobacter* species depends on the presence of the PGC. Through the analysis of the KO ID annotations, six *Erythrobacter* species, *E. litoralis*, *E. longus*, *E. lutimaris*, *E. marinus*, *E. odishensis*, and *E. zhengii*, were found to have both PGC and spheroidene/sprilloxanthin pathways required for the production of photosynthetic pigments (Fig. 5B and Table S5). The discovered PGC includes *puf*, encoding light-harvesting complex subunits; *bch*, for bacteriochlorophyll production; *chl*, for chlorophyll production; and *crt*, for additional carotenoid biosynthesis. A photosynthesis regulator, *ppsR*, is also present in the cluster and regulates the transcription of gene clusters such as that of *bch*, *crt*, *puf*, and *puc* [34]. As no PGC or additional carotenoid biosynthetic pathways were found in *Erythrobacter* sp. 3-20A1M, it is thought to be a heterotrophic bacterium and not an AAPB.

An evolutionary explanation for the lack of PGCs in *Erythrobacter* sp. 3-20A1M is that the presence of a large gene cluster is more likely to arise from a common ancestor than individual strains experiencing separate gene cluster deletions. However, in the phylogenetic analysis based on the UBCG, the AAPBs with PGCs did not appear to be bound under the common ancestral phylogeny branch (Fig. 1C). Although the UBCG analysis uses more sequences than the 16S rRNA sequence analysis, it remains a comparison using selected sets of genes. Since the absence of a large genetic element, such as a PGC, which is not included in the UBCG, may affect the analysis, phylogeny was re-analyzed by comparing the average nucleotide identity using the whole genome sequences (Fig. 5C) [35]. However, AAPB-possessing species were not grouped under the common ancestral branch, even in whole-genome sequence-based calculations. For example, the non-AAPB strain, *Erythrobacter* sp. 3-20A1M, is clustered near the AAPB strain, *E. lutimaris*. A more in-depth evolutionary analysis is needed to determine the evolutionary events that trigger the current distribution of PGCs across *Erythrobacter* species.

In summary, the complete genome sequence of a recently isolated marine bacterium, *Erythrobacter* sp. 3-20A1M, was assembled. Subsequent genotypic and pan-genome analyses were conducted to identify the bacterium. The comparative genomic analysis of the bacterium compared to other *Erythrobacter* species highlighted its inability to generate energy phototrophically and heterotrophic nature. Additionally, the presence of the virulence factors composed of biofilm generation, secretion system, flagella motility, and plant-algal cell wall degradation suggests its marine environmental niche as an active scavenger.

## Supplemental Materials



Supplementary data for this paper are available on-line only at http://jmb.or.kr.

## Figures and Tables

**Table 1:** Gene annotation statistics.

Features annotated	*Erythrobacter* sp. 3-20A1M
Coding sequences (CDSs)	2,946
Functional CDSs	2,830
Pseudogenes	116
RNA genes	52
rRNAs	1, 1, 1 (5S, 16S, 23S)
tRNAs	45
ncRNAs	4
Total annotated features	2,998

**Table 2:** *Erythrobacter* sp. specific genes regarding cell motility and signal transduction.

Cell motility			

Flagella assembly			

KEGG	Gene ID	Function	Locus tag

K02396	*flgK*	Flagellar hook-associated protein 1	F7D01_10300
K02398	*flgM*	Negative regulator of flagellin synthesis	F7D01_10230
K02416	*fliM*	Flagellar motor switch protein	F7D01_10160
K02422	*fliS*	Flagellar secretion chaperone	F7D01_10200
K02557	*motB*	Chemotaxis protein	F7D01_13825

Bacterial chemotaxis

KEGG	Gene ID	Function	Locus tag

K00575	*cheR*	Chemotaxis protein methyltransferase	F7D01_01680, F7D01_11905
K02416	*fliM*	Flagellar motor switch protein	F7D01_10160
K02557	*motB*	Chemotaxis protein	F7D01_13825
K03407	*cheA*	Chemotaxis sensor kinase	F7D01_11900
K03412	*cheB*	Protein-glutamate methylesterase/glutaminase	F7D01_01705, F7D01_01685, F7D01_11910
K13924	*cheR*	Chemotaxis protein methyltransferase	F7D01_04690

Signal transduction

Two-component system

KEGG	Gene ID	Function	Locus tag

K00405	*ccoO*	Cytochrome c oxidase cbb3-type subunit II	F7D01_07745
K00575	*cheR*	Chemotaxis protein methyltransferase	F7D01_01680, F7D01_11905
K01051	*pme*	Pectinesterase	F7D01_10375
K01104	*epsP*	Protein-tyrosine phosphatase	F7D01_13155
K01991	*epsA*	Polysaccharide biosynthesis/export protein	F7D01_05380
K02398	*flgM*	Negative regulator of flagellin synthesis	F7D01_10230
K02488	*pleD*	Cell cycle response regulator	F7D01_11555
K02659	*pilI*	Twitching motility protein PilI	F7D01_01700
K03407	*cheA*	Chemotaxis sensor kinase	F7D01_11900
K03412	*cheB*	Protein-glutamate methylesterase/glutaminase	F7D01_01705, F7D01_01685, F7D01_11910
K07165	*fecR*	Transmembrane sensor	F7D01_09650, F7D01_10520
K07782	*sdiA*	Quorum-sensing system regulator	F7D01_01835
K12340	*tolC*	Outer membrane protein	F7D01_05315
K13486	*wspC*	Chemotaxis protein methyltransferase	F7D01_02045, F7D01_03085
K13924	*cheR*	Chemotaxis protein methyltransferase	F7D01_04690
K18326	*mdtD*	Multidrug resistance protein	F7D01_04925
