# Supplementary material for: Assessment of Erythrobacter Species Diversity through Pan-Genome Analysis with Newly Isolated Erythrobacter sp. 3-20A1M
Source: J Microbiol Biotechnol. 2021 Feb 3;31(4):601–9. doi: 10.4014/jmb.2012.12054 (PMC9723273; doi:10.4014/jmb.2012.12054)
Supplement: Supplementary file 1 [file jmb-31-4-601-supple.pdf]

J. Microbiol. Biotechnol.
https://doi.org/10.4014/jmb.2012.12054

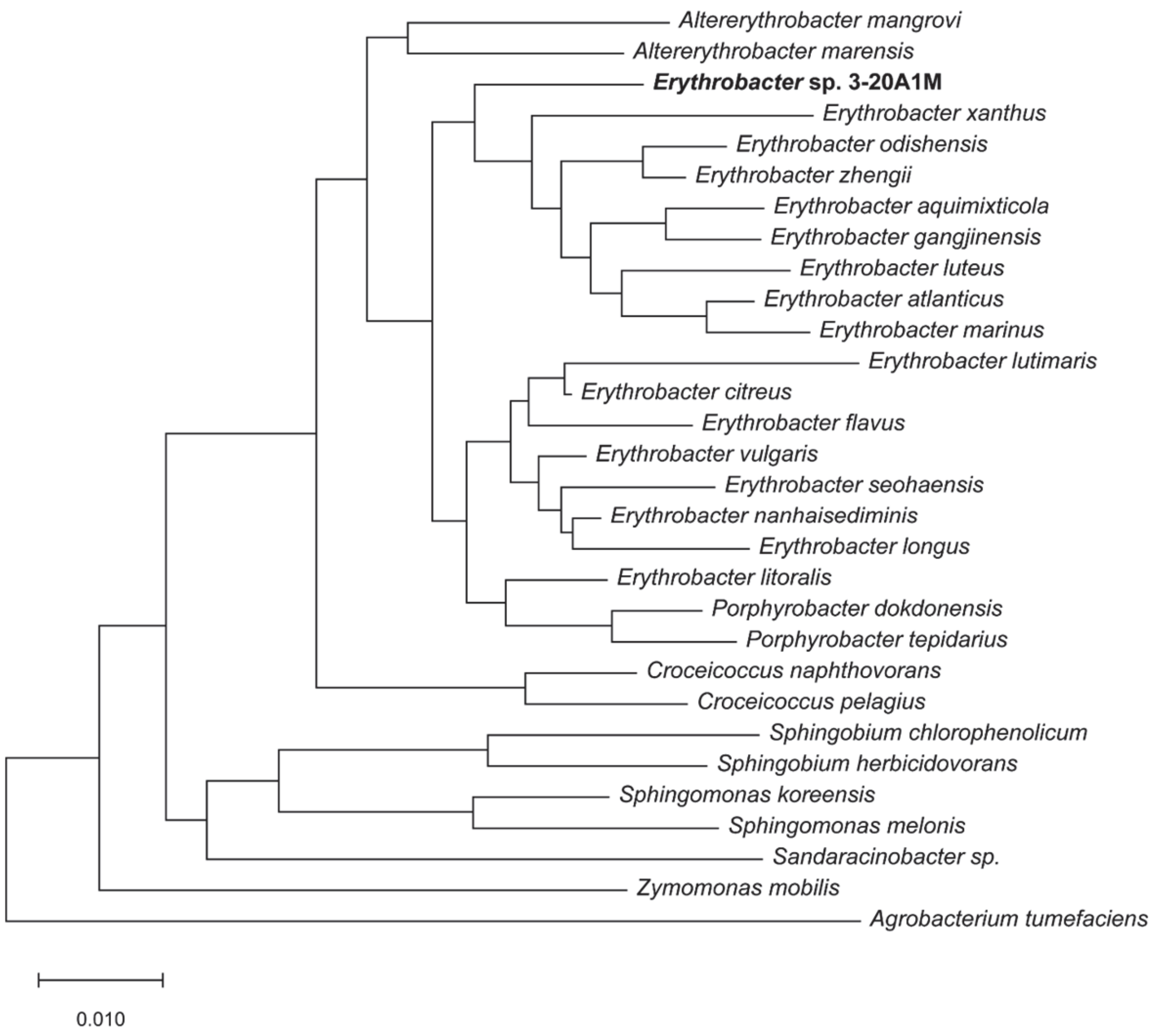

Supplementary Figure S1. 16s rRNA phylogenetic tree. The evolutionary history was inferred using the Neighbor-Joining method. The tree is drawn to scale, with branch lengths in the same units as those of the evolutionary distances used to infer the phylogenetic tree. The evolutionary distances were computed using the p-distance method and are in the units of the number of base differences per site. Evolutionary analyses were conducted in MEGA X.

Pan-genome analysis of *Erythrobacter* sp. 3-20A1M

Supplementary Table S1. Genome data used in this study

Species	Strain	Assembly	Note
Erythrobacter aquimixticola	JSSK-14	ASM360547v1	https://www.ncbi.nlm.nih.gov/assembly/GCF_003605475.1/
Erythrobacter atlanticus	s21-N3	ASM107781v2	https://www.ncbi.nlm.nih.gov/assembly/GCF_001077815.2/
Erythrobacter citreus	LAMA 915	Erythrobacter citreus LAMA 915 v1	https://www.ncbi.nlm.nih.gov/assembly/GCF_001235865.1/
Erythrobacter flavus	VG1	ASM223761v1	https://www.ncbi.nlm.nih.gov/assembly/GCF_002237615.1/
Erythrobacter gangjinensis	CGMCC1.15024	ASM188669v1	https://www.ncbi.nlm.nih.gov/assembly/GCF_001886695.1/
Erythrobacter litoralis	DSM 8509	ASM171916v1	https://www.ncbi.nlm.nih.gov/assembly/GCF_001719165.1/
Erythrobacter longus	DSM 6997	Ery_DSM 6997_v1	https://www.ncbi.nlm.nih.gov/assembly/GCF_000715015.1/
Erythrobacter luteus	KA37	ASM101094v1	https://www.ncbi.nlm.nih.gov/assembly/GCF_001010945.1/
Erythrobacter lutimaris	S-5	ASM336313v1	https://www.ncbi.nlm.nih.gov/assembly/1855411
Erythrobacter marinus	KCTC 23554	ASM102155v1	https://www.ncbi.nlm.nih.gov/assembly/GCF_001021555.1/
Erythrobacter nanhaisediminis	CGMCC 1.7715	IMG-taxon 2619618819 annotated assembly	https://www.ncbi.nlm.nih.gov/assembly/GCF_900115585.1/
Erythrobacter odishensis	KCTC 23981	ASM360519v1	https://www.ncbi.nlm.nih.gov/assembly/GCF_003605195.1/
Erythrobacter seohaensis	SW-135	ASM279586v1	https://www.ncbi.nlm.nih.gov/assembly/GCF_002795865.1/
Erythrobacter vulgaris	O1	IDBA-UD	https://www.ncbi.nlm.nih.gov/assembly/GCF_000756795.1/
Erythrobacter xanthus	CCTCC AB 2015396	ASM358401v1	https://www.ncbi.nlm.nih.gov/assembly/GCF_003584015.1/
Erythrobacter zhengii	V18	ASM358412v1	https://www.ncbi.nlm.nih.gov/assembly/GCF_003584125.1/
Agrobacterium tumefaciens	MAFF210266	ASM700286v1	https://www.ncbi.nlm.nih.gov/assembly/GCF_007002865.1
Altererythrobacter mangrovi	C9-11	ASM226934v1	https://www.ncbi.nlm.nih.gov/assembly/GCF_002269345.1/
Altererythrobacter marensis	KCTC 22370	ASM102862v1	https://www.ncbi.nlm.nih.gov/assembly/GCF_001028625.1
Croceicoccus naphthovorans	PQ-2	ASM102870v1	https://www.ncbi.nlm.nih.gov/assembly/GCF_001028705.1
Croceicoccus pelagius	Ery9	ASM166191v1	https://www.ncbi.nlm.nih.gov/assembly/GCF_001661915.1
Porphyrobacter dokdonensis	DSW-74	ASM167733v1	https://www.ncbi.nlm.nih.gov/assembly/GCF_001677335.1
Porphyrobacter tepidarius	DSM 10594	ASM215569v1	https://www.ncbi.nlm.nih.gov/assembly/GCF_002155695.1/
Sandaracinobacter	PAMC 28131	ASM638587v1	https://www.ncbi.nlm.nih.gov/assembly/GCF_006385875.1
Sphingobium chlorophenolicum	L-1	ASM14783v3	https://www.ncbi.nlm.nih.gov/assembly/GCF_000147835.2
Sphingobium herbicidovorans	MH	ASM208043v1	https://www.ncbi.nlm.nih.gov/assembly/GCF_002080435.1
Sphingomonas koreensis	ABOJV	ASM192238v1	https://www.ncbi.nlm.nih.gov/assembly/GCF_001922385.1
Sphingomonas melonis	ZJ26	ASM250426v1	https://www.ncbi.nlm.nih.gov/assembly/GCF_002504265.1
Zymomonas mobilis	ZM4	ASM710v1	https://www.ncbi.nlm.nih.gov/assembly/GCF_000007105.1

Supplementary Table S2. BUSCO genome assembly assessment.

**Strain**		***Erythrobacter* sp. 3-20A1M**		***Erythrobacter xanthus***	
BUSCO result		Count	Ratio (%)	Count	Ratio (%)
Complete (C)	Single-copy (S)	143	96.6	139	93.9
	Duplicated (D)	0	0	1	0.7
Fragmented (F)		0	0	0	0
Missing (M)		5	3.4	8	5.4
Total		148	100	148	100

Pan-genome analysis of *Erythrobacter* sp. 3-20A1M

Supplementary Table S3. Virulence factors of *Erythrobacter* sp. 3-20A1M.

Locus tag	Annotation	Gene name	Note
	**Biofilm**		
F7D01_05380	capsular biosynthesis protein	*epsA*	
F7D01_13520	UDP-N-acetylglucosamine 2-epimerase (non-hydrolyzing)	*epsC*	
F7D01_13155	protein-tyrosine-phosphatase	*epsP*	
F7D01_09625	polysaccharide deacetylase family protein	*pgdA*	
F7D01_05290	exopolysaccharide biosynthesis polyprenyl glycosylphosphotransferase	*pslA*	
F7D01_13545	polysaccharide biosynthesis tyrosine autokinase	*vpsO*	
F7D01_05365	polysaccharide pyruvyl transferase family protein	*wcaK*	
F7D01_10440	polysaccharide biosynthesis protein	*wza*	
F7D01_13540	polysaccharide export protein	*wza*	
F7D01_05355	exopolysaccharide biosynthesis protein		
F7D01_05390	diutan polysaccharide export protein		
	**Flagella**		
F7D01_10115	AAA domain-containing protein	*flaM*	
F7D01_10235	flagellar basal body P-ring formation protein FlgA	*flgA*	
F7D01_10285	flagellar basal body P-ring protein FlgI	*flgI*	
F7D01_10250	flagellar basal body rod protein FlgB	*flgB*	
F7D01_10255	flagellar basal body rod protein FlgC	*flgC*	
F7D01_10155	flagellar basal body-associated protein FliL	*fliL*	
F7D01_10275	flagellar basal-body rod protein FlgG	*flgG*	
F7D01_10170	flagellar biogenesis protein	*fliO*	
F7D01_10230	flagellar biosynthesis anti-sigma factor FlgM	*flgM*	
F7D01_10260	flagellar biosynthesis protein FlgD	*flgD*	
F7D01_10280	flagellar biosynthesis protein FlgH	*flgH*	
F7D01_10290	flagellar biosynthesis protein FlgJ	*flgJ*	
F7D01_10305	flagellar biosynthesis protein FlgL	*flgL*	
F7D01_10180	flagellar biosynthesis protein FliQ	*fliQ*	
F7D01_10185	flagellar biosynthetic protein FliR	*fliR*	
F7D01_10195	flagellar filament capping protein FliD	*fliD*	
F7D01_10295	flagellar hook-associated protein FlgK	*flgK*	
F7D01_10265	flagellar hook-basal body complex protein	*flgE*	
F7D01_10270	flagellar hook-basal body complex protein	*flgG*	
F7D01_10120	flagellar hook-basal body complex protein FliE	*fliE*	
F7D01_10310	flagellar motor stator protein MotA	*motA*	
F7D01_10130	flagellar motor switch protein FliG	*fliG*	
F7D01_10165	flagellar motor switch protein FliN	*fliN*	
F7D01_10125	flagellar M-ring protein FliF	*fliF*	
F7D01_10225	flagellar protein FlgN	*flgN*	
F7D01_10175	flagellar type III secretion system pore protein FliP	*fliP*	
F7D01_10215	flagellar type III secretion system protein FlhA	*flhA*	
F7D01_10190	flagellar type III secretion system protein FlhB	*flhB*	
F7D01_10110	flagellin FliC	*fliC*	
F7D01_10140	FliI/YscN family ATPase	*fliI*	
F7D01_10300	hypothetical protein	*flgK*	
F7D01_10135	hypothetical protein	*fliH*	
F7D01_10160	hypothetical protein	*fliM*	
F7D01_10200	hypothetical protein	*fliS*	
F7D01_10245	hypothetical protein	*motA*	

F7D01_13825	hypothetical protein	*motB*	
F7D01_10315	OmpA family protein	*motB*	
F7D01_08885	RNA polymerase factor sigma-54	*rpoN*	
F7D01_00160	RNA polymerase sigma factor RpoD	*rpoD*	
F7D01_10210	sigma-70 family RNA polymerase sigma factor	*fliA*	
	**Secretion system**		
F7D01_05320	type I secretion system permease/ATPase	*eexD*	Type I
F7D01_05315	TolC family outer membrane protein	*tolC*	
F7D01_06450	TolC family outer membrane protein	*tolC*	
F7D01_03245	type II secretion system F family protein	*tadC*	Type II
F7D01_10215	flagellar type III secretion system protein FlhA	*flhA*	Type III
F7D01_10190	flagellar type III secretion system protein FlhB	*flhB*	
F7D01_10175	flagellar type III secretion system pore protein FliP	*fliP*	
F7D01_09320	type IV secretion system protein TraC	*traC*	Type IV
F7D01_03700	type VI secretion protein	*virB10*	
F7D01_03695	P-type DNA transfer ATPase VirB11	*virB11*	
F7D01_03725	type VI secretion protein	*virB2*	
F7D01_03720	type VI secretion protein	*virB3*	
F7D01_03715	VirB4 family type IV secretion/conjugal transfer ATPase	*virB4*	
F7D01_03710	type VI secretion protein	*virB6*	
F7D01_03705	type VI secretion protein	*virB9*	
F7D01_12395	type IV secretion system DNA-binding domain-containing protein		
F7D01_03815	hypothetical protein	*yadA*	Type Vc
F7D01_14775	vgr related protein	*vgrG*	Type VI
F7D01_12175	signal recognition particle-docking protein FtsY	*ftsY*	Sec-SRP
F7D01_13480	preprotein translocase subunit SecA	*secA*	
F7D01_08305	protein-export chaperone SecB	*secB*	
F7D01_11600	protein translocase subunit SecD	*secD*	
F7D01_04655	preprotein translocase subunit SecE	*secE*	
F7D01_11605	protein translocase subunit SecF	*secF*	
F7D01_14405	preprotein translocase subunit SecG	*secG*	
F7D01_01380	preprotein translocase subunit SecY	*secY*	
F7D01_00885	signal recognition particle protein	*srp54*	
F7D01_02415	tRNA (adenosine(37)-N6)-threonylcarbamoyltransferase complex transferase subunit TsaD	*tsaD*	
F7D01_11595	preprotein translocase subunit YajC	*yajC*	
F7D01_05115	membrane protein insertase YidC	*yidC*	
F7D01_03155	twin-arginine translocase TatA/TatE family subunit	*tatA*	Tat
F7D01_03150	twin-arginine translocase subunit TatB	*tatB*	
F7D01_03145	twin-arginine translocase subunit TatC	*tatC*	
F7D01_03225	secretion system protein	*cpaC*	
	**Plant-algae cell wall degradation**		
F7D01_10565	cellulase family glycosylhydrolase	*bgaB*	
F7D01_10535	family 1 glycosylhydrolase	*bglB*	
F7D01_10540	family 1 glycosylhydrolase	*bglB*	
F7D01_09485	1, 4-beta-D-glucan glucohydrolase	*bglX*	
F7D01_10905	beta-glucosidase BglX	*bglX*	
F7D01_13025	beta-glucosidase	*bglX*	
F7D01_13030	beta-glucosidase	*bglX*	
F7D01_13095	1, 4-beta-D-glucan glucohydrolase	*bglX*	
F7D01_06485	cell wall hydrolase	*cwlJ*	

Pan-genome analysis of *Erythrobacter* sp. 3-20A1M

F7D01_05925	UTP--glucose-1-phosphate uridylyltransferase GalU	*galU*
F7D01_04500	glucokinase	*glk*
F7D01_13105	ROK family protein	*glk*
F7D01_04785	DUF3459 domain-containing protein	*malZ*
F7D01_12500	glycoside hydrolase family 65 protein	*mapA*
F7D01_10545	alpha/beta hydrolase fold domain-containing protein	*mlhB*
F7D01_10550	alpha/beta hydrolase fold domain-containing protein	*mlhB*
F7D01_13150	trehalose-6-phosphate synthase	*otsA*
F7D01_13140	trehalose-phosphatase	*otsB*
F7D01_10345	pectate lyase	*pelL*
F7D01_13280	glucose-6-phosphate isomerase	*pgi*
F7D01_09690	6-phosphogluconolactonase	*pgi*
F7D01_05410	alpha-D-glucose phosphate-specific phosphoglucomutase	*pgm*
F7D01_07065	phosphomannomutase	*pgm*
F7D01_10375	alpha/beta hydrolase fold domain-containing protein	*pme*
F7D01_06300	adenosine kinase	*scrK*
F7D01_13145	glycoside hydrolase family 15 protein	*sga1*
F7D01_11430	HAD-IIB family hydrolase	*spp*
F7D01_01595	TlyA family rRNA (cytidine-2'-O)-methyltransferase	*tlyA*
F7D01_10570	alpha, alpha-trehalase TreF	*treF*
F7D01_12505	trehalose-phosphatase	*treF*
F7D01_10530	alpha/beta fold hydrolase	
F7D01_10555	hypothetical protein	
F7D01_13285	PEP-CTERM sorting domain-containing protein	
F7D01_04780	glycoside hydrolase family 97 protein	
F7D01_10915	glycoside hydrolase family 97 protein	
F7D01_10360	TRAP transporter small permease subunit	*dctQ*
F7D01_10355	DctP family TRAP transporter solute-binding subunit	*yiaO*

Supplementary Table S4. AntiSMASH prediction of secondary metabolites biosynthetic gene clusters

#	Strain	Contig	Region	Type	From	To	Most similar known cluster	Similarity
1	*Erythrobacter* sp. 3-20A1M	CP045200	Region 1	T3PKS	315,403	356,446		
		CP045200	Region 2	lassopeptide,hserlactone	1,649,973	1,687,811		
		CP045200	Region 3	terpene	1,984,024	2,008,298	zeaxanthin	66%
2	*Erythrobacter aquimixticola*	NZ_RAHX01000001	Region 1.1	T3PKS	1,032,494	1,073,561		
		NZ_RAHX01000001	Region 1.2	terpene	2,334,005	2,358,384		
3	*Erythrobacter flavus*	NZ_CP022528.1	Region 1	terpene	2,713,680	2,738,023	carotenoid	33%
		NZ_CP022528.1	Region 2	bacteriocin	3,023,136	3,033,975		
4	*Erythrobacter atlanticus*	NZ_CP011310.1	Region 1.1	lassopeptide	257,946	280,508	S-layer glycan	20%
		NZ_CP011310.1	Region 1.2	betalactone	606,681	637,153		
		NZ_CP011310.1	Region 1.3	T3PKS	1,532,661	1,573,740		
		NZ_CP011310.1	Region 1.4	terpene	2,643,212	2,667,666		
5	*Erythrobacter citreus* LAMA 915	JYNE01000026.1	Region 8.1	terpene	237,062	257,823		
		JYNE01000028.1	Region 13.1	hserlactone	396,047	416,616		
6	*Erythrobacter gangjinensis*	NZ_CP018097.1	Region 1.1	lassopeptide	489,588	512,943	S-layer glycan	20%
		NZ_CP018097.1	Region 1.2	terpene	1,147,944	1,172,816	zeaxanthin	66%
		NZ_CP018097.1	Region 1.3	T3PKS	1,610,817	1,651,878		
7	*Erythrobacter litoralis*	CP017057.1	Region 1	T1PKS,NRPS-like	415,708	467,897	lipopolysaccharide	16%
		CP017057.1	Region 2	terpene	752,393	776,822	carotenoid	16%
8	*Erythrobacter longus*	NZ_JMIW01000001	Region 1.1	terpene	1,207,151	1,232,225	carotenoid	16%
		NZ_JMIW01000009	Region 9.1	bacteriocin	43,588	54,445		
		NZ_JMIW01000009	Region 9.2	lassopeptide	129,036	151,467	S-layer glycan	20%
9	*Erythrobacter luteus*	NZ_LBHB01000001	Region 1.1	lassopeptide	186,723	209,331		
		NZ_LBHB01000001	Region 1.2	T3PKS	1,086,616	1,127,713		
		NZ_LBHB01000002	Region 11.1	terpene	990,801	1,015,707	zeaxanthin	66%
10	*Erythrobacter nanhaisediminis*	NZ_FOWZ01000001	Region 5.1	terpene	973,100	997,416	zeaxanthin	66%
11	*Erythrobacter vulgaris*	NZ_CCSI01000003	Region 3.1	terpene	264,281	288,621	zeaxanthin	66%
12	*Erythrobacter lutimaris*	NZ_QRBB01000001	Region 4.1	terpene	94,452	119,258	zeaxanthin	66%
		NZ_QRBB01000001	Region 4.2	lassopeptide	672,213	701,326	capsular polysaccharide	3%
		NZ_QRBB01000001	Region 4.3	bacteriocin, lanthipeptide	1,194,495	1,218,692		
13	*Erythrobacter seohaensis*	NZ_CP024920.1	Region 1	lassopeptide	440,381	462,679		

Pan-genome analysis of *Erythrobacter* sp. 3-20A1M

		NZ_CP024920.1	Region 2	bacteriocin	1,859,624	1,870,472		
		NZ_CP024920.1	Region 3	terpene	1,993,945	2,018,261	zeaxanthin	66%
14	*Erythrobacter odishensis*	NZ_QYOS01000025	Region 11.1	T3PKS	252,492	293,559		
		NZ_QYOS01000024	Region 18.1	terpene	295,716	320,144		
		NZ_QYOS01000023	Region 19.1	lassopeptide	412,290	435,012	S-layer glycan	20%
15	*Erythrobacter marinus*	NZ_LDCP01000001	Region 1.1	lassopeptide	572,157	595,561	S-layer glycan	20%
		NZ_LDCP01000002	Region 2.1	T3PKS	471,232	512,296		
		NZ_LDCP01000003	Region 3.1	lassopeptide	35,615	59,222		
		NZ_LDCP01000004	Region 4.1	terpene	263,740	288,170		
16	*Erythrobacter zhengii* V18	NZ_QXFL01000001	Region 1.1	T3PKS	468,525	509,592		
		NZ_QXFL01000019	Region 11.1	betalactone	1	17,144		
		NZ_QXFL01000002	Region 12.1	betalactone	609,812	641,208	lipopolysaccharide	5%
		NZ_QXFL01000004	Region 24.1	lassopeptide	58,971	89,940		
		NZ_QXFL01000004	Region 24.2	terpene	196,548	220,991		
		NZ_QXFL01000009	Region 29.1	betalactone	1	25,330		
17	*Erythrobacter xanthus*	NZ_QXFM01000033	Region 20.1	terpene	1	4,548		
		NZ_QXFM01000005	Region 40.1	terpene	1	9,686	carotenoid	33%
		NZ_QXFM01000057	Region 45.1	lassopeptide	51,011	73,276		
		NZ_QXFM01000007	Region 68.1	lassopeptide,hserlactone	1	24,575	paulomycin	3%
		NZ_QXFM01000083	Region 74.1	lassopeptide	13,793	26,474		
		NZ_QXFM01000084	Region 75.1	NRPS	1	24,972		
		NZ_QXFM01000090	Region 81.1	NRPS-like	.	2,198	rhizomide A / rhizomide B / rhizomide C	100%
		NZ_QXFM01000091	Region 82.1	NRPS	1	2,288		
		NZ_QXFM01000096	Region 87.1	lassopeptide	7,841	23,674		
		NZ_QXFM01000097	Region 89.1	NRPS	1	8,516		
		NZ_QXFM01000098	Region 90.1	NRPS	1	2,288		
		NZ_QXFM01000115	Region 108.1	NRPS-like	1	2,159		
		NZ_QXFM01000145	Region 138.1	NRPS	1	22,399	acyldepsipeptide 1	15%

Supplementary Table S5. Photosynthetic gene clusters of *Erythrobacter* species.

Strain	Contig	Start	End	F/R	Locus tag	Gene ID	Function
E. litoralis	CP017057	938351	939787	-	Ga0102493_111818	*pucC*	MFS transporter, BCD family, chlorophyll transporter
	CP017057	939784	940506	-	Ga0102493_111819	*bchM*	magnesium-protoporphyrin O-methyltransferase
	CP017057	940503	941399	-	Ga0102493_111820	*chlL*	light-independent protochlorophyllide reductase subunit L
	CP017057	941422	945072	-	Ga0102493_111821	*bchH*	magnesium chelatase subunit H
	CP017057	945062	946648	-	Ga0102493_111822	*chlB*	light-independent protochlorophyllide reductase subunit B
	CP017057	946652	947953	-	Ga0102493_111823	*chlN*	light-independent protochlorophyllide reductase subunit N
	CP017057	947950	948519	-	Ga0102493_111824	*bchF*	3-vinyl bacteriochlorophyllide hydratase
	CP017057	948792	949703	+	Ga0102493_111825	*btuF*	Methanogenic corrinoid protein MtbC1
	CP017057	949716	951140	+	Ga0102493_111826	*ppsR*	transcriptional regulator PpsR
	CP017057	951271	952173	+	Ga0102493_111827	*bchG*	chlorophyll synthase
	CP017057	952173	953516	+	Ga0102493_111828	*pucC*	MFS transporter, BCD family, chlorophyll transporter
	CP017057	953513	954709	+	Ga0102493_111829	*bchP*	geranylgeranyl reductase
	CP017057	954720	955202	+	Ga0102493_111830	*tspO*	tryptophan-rich sensory protein
	CP017057	955447	955701	+	Ga0102493_111831		hypothetical protein
	CP017057	955930	956853	-	Ga0102493_111832	*pufM*	photosynthetic reaction center M subunit
	CP017057	956868	957692	-	Ga0102493_111833	*pufL*	photosynthetic reaction center L subunit
	CP017057	957790	957984	-	Ga0102493_111834	*pufA*	light-harvesting complex 1 alpha chain
	CP017057	958053	958229	-	Ga0102493_111835	*pufB*	light-harvesting complex 1 beta chain
	CP017057	958386	959831	-	Ga0102493_111836	*bchZ*	chlorophyllide a reductase subunit Z
	CP017057	959828	961429	-	Ga0102493_111837	*bchY*	chlorophyllide a reductase subunit Y
	CP017057	961434	962423	-	Ga0102493_111838	*bchX*	chlorophyllide a reductase subunit X
	CP017057	962420	963355	-	Ga0102493_111839	*bchC*	3-hydroxyethyl bacteriochlorophyllide a dehydrogenase
	CP017057	963487	964608	-	Ga0102493_111840	*crtF*	demethylspheroidene O-methyltransferase
	CP017057	964693	966285	+	Ga0102493_111841	*crtD*	1-hydroxycarotenoid 3,4-desaturase
	CP017057	966405	967124	+	Ga0102493_111842	*crtC*	carotenoid 1,2-hydratase
	CP017057	967134	967286	-	Ga0102493_111843		hypothetical protein
	CP017057	967283	968176	-	Ga0102493_111844	*bchO*	magnesium chelatase accessory protein
	CP017057	968173	969984	-	Ga0102493_111845	*bchD*	magnesium chelatase subunit D

Pan-genome analysis of *Erythrobacter* sp. 3-20A1M

	CP017057	969981	970982	-	Ga0102493_111846	*bchI*	magnesium chelatase subunit I
	CP017057	971081	972781	-	Ga0102493_111847	*sfuB*	iron(III) transport system permease protein
	CP017057	2771973	2774057	-	Ga0102493_11470		TolB amino-terminal domain-containing protein
	CP017057	2774432	2775124	+	Ga0102493_11471		ubiquinol oxidase
	CP017057	2775180	2776211	-	Ga0102493_11472	*bchJ*	bacteriochlorophyll 4-vinyl reductase
	CP017057	2776201	2777829	-	Ga0102493_11473	*bchE*	anaerobic magnesium-protoporphyrin IX monomethyl ester cyclase
E. longus	NZ_JMIW01000003	138241	139635	-	EH31_RS07900	*pucC*	MFS transporter
	NZ_JMIW01000003	139671	140393	-	EH31_RS07905	*bchM*	magnesium protoporphyrin IX methyltransferase
	NZ_JMIW01000003	140393	141289	-	EH31_RS07910	*chlL*	ferredoxin:protochlorophyllide reductase (ATP-dependent) iron-sulfur ATP-binding protein
	NZ_JMIW01000003	141306	144941	-	EH31_RS07915	*bchH*	magnesium chelatase subunit H
	NZ_JMIW01000003	144931	146496	-	EH31_RS07920	*chlB*	ferredoxin:protochlorophyllide reductase (ATP-dependent) subunit B
	NZ_JMIW01000003	146499	147794	-	EH31_RS07925	*chlN*	ferredoxin:protochlorophyllide reductase (ATP-dependent) subunit N
	NZ_JMIW01000003	147791	148366	-	EH31_RS07930	*bchF*	2-vinyl bacteriochlorophyllide hydratase
	NZ_JMIW01000003	148865	149461	+	EH31_RS07935	*btuF*	cobalamin B12-binding protein
	NZ_JMIW01000003	149474	150898	+	EH31_RS07940	*ppsR*	transcriptional regulator PpsR
	NZ_JMIW01000003	151166	152131	+	EH31_RS07945	*bchG*	chlorophyll synthase ChlG
	NZ_JMIW01000003	152131	153519	+	EH31_RS07950	*pucC*	BCD family MFS transporter
	NZ_JMIW01000003	153516	154739	+	EH31_RS07955	*bchP*	geranylgeranyl diphosphate reductase
	NZ_JMIW01000003	154797	155327	+	EH31_RS07960	*tspO*	tryptophan-rich sensory protein
	NZ_JMIW01000003	155349	155603	+	EH31_RS07965		hypothetical protein
	NZ_JMIW01000003	155708	158071	-	EH31_RS07970	*susC*	TonB-dependent receptor
	NZ_JMIW01000003	158299	159225	-	EH31_RS07975	*pufM*	photosynthetic reaction center subunit M
	NZ_JMIW01000003	159225	160061	-	EH31_RS07980	*pufL*	photosynthetic reaction center subunit L
	NZ_JMIW01000003	160196	160324	-	EH31_RS17215	*pufA*	light-harvesting protein
	NZ_JMIW01000003	160411	160569	-	EH31_RS17220	*pufB*	light-harvesting protein
	NZ_JMIW01000003	160720	162204	-	EH31_RS07985	*bchZ*	chlorophyllide a reductase subunit Z
	NZ_JMIW01000003	162201	163649	-	EH31_RS07990	*bchY*	chlorophyllide a reductase subunit Y
	NZ_JMIW01000003	163768	164757	-	EH31_RS07995	*bchX*	chlorophyllide a reductase iron protein subunit X
	NZ_JMIW01000003	164754	165689	-	EH31_RS08000	*bchC*	chlorophyll synthesis pathway protein BchC
	NZ_JMIW01000003	165850	166983	-	EH31_RS08005	*crtF*	methyltransferase domain-containing protein
	NZ_JMIW01000003	167104	168684	+	EH31_RS08010	*crtD*	phytoene desaturase

	NZ_JMIW01000003	168798	169517	+	EH31_RS08015	*crtC*	hydroxyneurosporene dehydrogenase
	NZ_JMIW01000003	169514	169732	-	EH31_RS08020		hypothetical protein
	NZ_JMIW01000003	169729	170634	-	EH31_RS08025	*bchO*	alpha/beta fold hydrolase
	NZ_JMIW01000003	170631	172307	-	EH31_RS08030	*bchD*	magnesium chelatase subunit D
	NZ_JMIW01000003	172304	173305	-	EH31_RS08035	*bchI*	magnesium chelatase ATPase subunit I
	NZ_JMIW01000003	173390	175066	-	EH31_RS08040	*sfuB*	iron ABC transporter permease
	NZ_QRBB01000001	1234761	1235141	-	DL238_RS06040		VOC family protein
	NZ_QRBB01000001	1235255	1236253	+	DL238_RS06045	*bchI*	magnesium chelatase ATPase subunit I
	NZ_QRBB01000001	1236253	1237947	+	DL238_RS06050	*bchD*	magnesium chelatase subunit D
	NZ_QRBB01000001	1237944	1238822	+	DL238_RS06055	*bchO*	alpha/beta fold hydrolase
	NZ_QRBB01000001	1238979	1239407	+	DL238_RS06060		gamma-glutamylcyclotransferase
	NZ_QRBB01000001	1239439	1240158	-	DL238_RS06065	*crtC*	hydroxyneurosporene dehydrogenase
	NZ_QRBB01000001	1240278	1241864	-	DL238_RS06070	*crtD*	phytoene desaturase
	NZ_QRBB01000001	1241975	1243057	+	DL238_RS06075	*crtF*	methyltransferase domain-containing protein
	NZ_QRBB01000001	1243266	1244201	+	DL238_RS06080	*bchC*	chlorophyll synthesis pathway protein BchC
	NZ_QRBB01000001	1244198	1245193	+	DL238_RS06085	*bchX*	chlorophyllide a reductase iron protein subunit X
	NZ_QRBB01000001	1245198	1246739	+	DL238_RS06090	*bchY*	chlorophyllide a reductase subunit Y
	NZ_QRBB01000001	1246906	1248366	+	DL238_RS06095	*bchZ*	chlorophyllide a reductase subunit Z
E. lutimaris	NZ_QRBB01000001	1248491	1248655	+	DL238_RS06100	*pufB*	light-harvesting protein
	NZ_QRBB01000001	1248733	1248921	+	DL238_RS06105	*pufA*	light-harvesting protein
	NZ_QRBB01000001	1249050	1249874	+	DL238_RS06110	*pufL*	photosynthetic reaction center subunit L
	NZ_QRBB01000001	1249888	1250811	+	DL238_RS06115	*pufM*	photosynthetic reaction center subunit M
	NZ_QRBB01000001	1250882	1251082	-	DL238_RS06120		hypothetical protein
	NZ_QRBB01000001	1251141	1251620	-	DL238_RS06125	*tspO*	tryptophan-rich sensory protein
	NZ_QRBB01000001	1251648	1252841	-	DL238_RS06130	*bchP*	geranylgeranyl diphosphate reductase
	NZ_QRBB01000001	1252838	1254175	-	DL238_RS06135	*pucC*	MFS transporter
	NZ_QRBB01000001	1254175	1255077	-	DL238_RS06140	*bchG*	chlorophyll synthase ChlG
	NZ_QRBB01000001	1255144	1256562	-	DL238_RS06145	*ppsR*	transcriptional regulator PpsR
	NZ_QRBB01000001	1256577	1257323	-	DL238_RS06150	*btuF*	cobalamin B12-binding protein
	NZ_QRBB01000001	1257676	1258215	+	DL238_RS06155	*bchF*	2-vinyl bacteriochlorophyllide hydratase
	NZ_QRBB01000001	1258212	1259507	+	DL238_RS06160	*chlN*	ferredoxin:protochlorophyllide reductase (ATP-dependent) subunit N

	NZ_QRBB01000001	1259510	1261138	+	DL238_RS06165	*chlB*	ferredoxin:protochlorophyllide reductase (ATP-dependent) subunit B
	NZ_QRBB01000001	1261128	1264670	+	DL238_RS06170	*bchH*	magnesium chelatase subunit H
	NZ_QRBB01000001	1264681	1265568	+	DL238_RS06175	*chlL*	ferredoxin:protochlorophyllide reductase (ATP-dependent) iron-sulfur ATP-binding protein
	NZ_QRBB01000001	1265568	1266293	+	DL238_RS06180	*bchM*	magnesium protoporphyrin IX methyltransferase
	NZ_QRBB01000001	1266290	1267720	+	DL238_RS06185	*pucC*	MFS transporter
E. marinus	NZ_LDCP01000004	33678	33998	-	AB731_RS12275		TrbC/VirB2 family protein
	NZ_LDCP01000004	34097	35098	+	AB731_RS12280	*bchI*	magnesium chelatase ATPase subunit I
	NZ_LDCP01000004	35095	36807	+	AB731_RS12285	*bchD*	magnesium chelatase subunit D
	NZ_LDCP01000004	36804	37691	+	AB731_RS12290	*bchO*	alpha/beta fold hydrolase
	NZ_LDCP01000004	37808	38527	-	AB731_RS12295	*crtC*	hydroxyneurosporene dehydrogenase
	NZ_LDCP01000004	38647	40212	-	AB731_RS12300	*crtD*	phytoene desaturase
	NZ_LDCP01000004	40293	41429	+	AB731_RS12305	*crtF*	methyltransferase domain-containing protein
	NZ_LDCP01000004	41546	42481	+	AB731_RS12310	*bchC*	chlorophyll synthesis pathway protein BchC
	NZ_LDCP01000004	42478	43464	+	AB731_RS12315	*bchX*	chlorophyllide a reductase iron protein subunit X
	NZ_LDCP01000004	43468	45015	+	AB731_RS12320	*bchY*	chlorophyllide a reductase subunit Y
	NZ_LDCP01000004	45012	46457	+	AB731_RS12325	*bchZ*	chlorophyllide a reductase subunit Z
	NZ_LDCP01000004	46578	46736	+	AB731_RS13860	*pufB*	light-harvesting protein
	NZ_LDCP01000004	46801	46968	+	AB731_RS13865	*pufA*	light-harvesting protein
	NZ_LDCP01000004	47095	47919	+	AB731_RS12330	*pufL*	photosynthetic reaction center subunit L
	NZ_LDCP01000004	47934	48911	+	AB731_RS12335	*pufM*	photosynthetic reaction center subunit M
	NZ_LDCP01000004	48908	49963	+	AB731_RS12340	*pufC*	photosynthetic reaction center cytochrome c subunit
	NZ_LDCP01000004	50037	50243	-	AB731_RS12345		hypothetical protein
	NZ_LDCP01000004	50319	50786	-	AB731_RS12350	*tspO*	tryptophan-rich sensory protein
	NZ_LDCP01000004	50804	52000	-	AB731_RS12355	*bchP*	geranylgeranyl diphosphate reductase
	NZ_LDCP01000004	51997	53316	-	AB731_RS12360	*pucC*	BCD family MFS transporter
	NZ_LDCP01000004	53313	54215	-	AB731_RS12365	*bchG*	chlorophyll synthase ChlG
	NZ_LDCP01000004	54285	55706	-	AB731_RS12370	*ppsR*	transcriptional regulator PpsR
	NZ_LDCP01000004	55720	56334	-	AB731_RS12375	*btuF*	cobalamin B12-binding protein
	NZ_LDCP01000004	56898	57404	+	AB731_RS12380	*bchF*	2-vinyl bacteriochlorophyllide hydratase
	NZ_LDCP01000004	57401	58696	+	AB731_RS12385	*chlN*	ferredoxin:protochlorophyllide reductase (ATP-dependent) subunit N
	NZ_LDCP01000004	58701	60236	+	AB731_RS12390	*chlB*	ferredoxin:protochlorophyllide reductase (ATP-dependent) subunit B

	NZ_LDCP01000004	60226	63813	+	AB731_RS12395	*bchH*	magnesium chelatase subunit H
	NZ_LDCP01000004	63823	64722	+	AB731_RS12400	*chlL*	ferredoxin:protochlorophyllide reductase (ATP-dependent) iron-sulfur ATP-binding protein
	NZ_LDCP01000004	64722	65453	+	AB731_RS12405	*bchM*	magnesium protoporphyrin IX methyltransferase
	NZ_LDCP01000004	65453	66880	+	AB731_RS12410	*pucC*	BCD family MFS transporter
E. odishensis	NZ_QYOS01000024	423580	425007	-	D4Q51_RS11375	*pucC*	MFS transporter
	NZ_QYOS01000024	425007	425708	-	D4Q51_RS11380	*bchM*	magnesium protoporphyrin IX methyltransferase
	NZ_QYOS01000024	425708	426604	-	D4Q51_RS11385	*chlL*	ferredoxin:protochlorophyllide reductase (ATP-dependent) iron-sulfur ATP-binding protein
	NZ_QYOS01000024	426618	430166	-	D4Q51_RS11390	*bchH*	magnesium chelatase subunit H
	NZ_QYOS01000024	430156	431685	-	D4Q51_RS11395	*chlB*	ferredoxin:protochlorophyllide reductase (ATP-dependent) subunit B
	NZ_QYOS01000024	431690	432988	-	D4Q51_RS11400	*chlN*	ferredoxin:protochlorophyllide reductase (ATP-dependent) subunit N
	NZ_QYOS01000024	432985	433515	-	D4Q51_RS11405	*bchF*	2-vinyl bacteriochlorophyllide hydratase
	NZ_QYOS01000024	433726	434622	+	D4Q51_RS11410	*btuF*	cobalamin B12-binding protein
	NZ_QYOS01000024	434634	436052	+	D4Q51_RS11415	*ppsR*	transcriptional regulator PpsR
	NZ_QYOS01000024	436118	437023	+	D4Q51_RS11420	*bchG*	chlorophyll synthase ChlG
	NZ_QYOS01000024	437031	438356	+	D4Q51_RS11425	*pucC*	MFS transporter
	NZ_QYOS01000024	438353	439549	+	D4Q51_RS11430	*bchP*	geranylgeranyl diphosphate reductase
	NZ_QYOS01000024	439546	440025	+	D4Q51_RS11435	*tspO*	tryptophan-rich sensory protein
	NZ_QYOS01000024	440086	440280	+	D4Q51_RS11440		hypothetical protein
	NZ_QYOS01000024	440291	441406	-	D4Q51_RS11445	*pufC*	photosynthetic reaction center cytochrome c subunit
	NZ_QYOS01000024	441403	442377	-	D4Q51_RS11450	*pufM*	photosynthetic reaction center subunit M
	NZ_QYOS01000024	442392	443216	-	D4Q51_RS11455	*pufL*	photosynthetic reaction center subunit L
	NZ_QYOS01000024	443315	443491	-	D4Q51_RS11460	*pufA*	light-harvesting protein
	NZ_QYOS01000024	443573	443728	-	D4Q51_RS11465	*pufB*	light-harvesting protein
	NZ_QYOS01000024	443847	445292	-	D4Q51_RS11470	*bchZ*	chlorophyllide a reductase subunit Z
	NZ_QYOS01000024	445289	446812	-	D4Q51_RS11475	*bchY*	chlorophyllide a reductase subunit Y
	NZ_QYOS01000024	446843	447841	-	D4Q51_RS11480	*bchX*	chlorophyllide a reductase iron protein subunit X
	NZ_QYOS01000024	447838	448773	-	D4Q51_RS11485	*bchC*	chlorophyll synthesis pathway protein BchC
	NZ_QYOS01000024	448882	450042	-	D4Q51_RS11490	*crtF*	methyltransferase domain-containing protein
	NZ_QYOS01000024	450108	451667	+	D4Q51_RS11495	*crtD*	phytoene desaturase
	NZ_QYOS01000024	451787	452503	+	D4Q51_RS11500	*crtC*	hydroxyneurosporene dehydrogenase

	NZ_QYOS01000024	452453	452638	-	D4Q51_RS11505		hypothetical protein
	NZ_QYOS01000024	452640	453545	-	D4Q51_RS11510	*bchO*	alpha/beta fold hydrolase
	NZ_QYOS01000024	453542	455215	-	D4Q51_RS11515	*bchD*	magnesium chelatase subunit D
	NZ_QYOS01000024	455223	456224	-	D4Q51_RS11520	*bchI*	magnesium chelatase ATPase subunit I
	NZ_QYOS01000024	456304	457671	-	D4Q51_RS11525		TolC family protein
E. zhengii	NZ_QXFL01000004	12125	12823	-	D2V07_RS09550		Crp/Fnr family transcriptional regulator
	NZ_QXFL01000004	12955	13956	+	D2V07_RS09555	*bchI*	magnesium chelatase ATPase subunit I
	NZ_QXFL01000004	13957	15645	+	D2V07_RS09560	*bchD*	magnesium chelatase subunit D
	NZ_QXFL01000004	15642	16559	+	D2V07_RS09565	*bchO*	alpha/beta fold hydrolase
	NZ_QXFL01000004	16552	16752	+	D2V07_RS09570		hypothetical protein
	NZ_QXFL01000004	16702	17418	-	D2V07_RS09575	*crtC*	hydroxyneurosporene dehydrogenase
	NZ_QXFL01000004	17538	19097	-	D2V07_RS09580	*crtD*	phytoene desaturase
	NZ_QXFL01000004	19212	20321	+	D2V07_RS09585	*crtF*	methyltransferase domain-containing protein
	NZ_QXFL01000004	20431	21366	+	D2V07_RS09590	*bchC*	chlorophyll synthesis pathway protein BchC
	NZ_QXFL01000004	21363	22361	+	D2V07_RS09595	*bchX*	chlorophyllide a reductase iron protein subunit X
	NZ_QXFL01000004	22401	23924	+	D2V07_RS09600	*bchY*	chlorophyllide a reductase subunit Y
	NZ_QXFL01000004	23921	25366	+	D2V07_RS09605	*bchZ*	chlorophyllide a reductase subunit Z
	NZ_QXFL01000004	25488	25643	+	D2V07_RS09610	*pufB*	light-harvesting protein
	NZ_QXFL01000004	25725	25898	+	D2V07_RS09615	*pufA*	light-harvesting protein
	NZ_QXFL01000004	26000	26824	+	D2V07_RS09620	*pufL*	photosynthetic reaction center subunit L
	NZ_QXFL01000004	26839	27813	+	D2V07_RS09625	*pufM*	photosynthetic reaction center subunit M
	NZ_QXFL01000004	27810	28925	+	D2V07_RS09630	*pufC*	photosynthetic reaction center cytochrome c subunit
	NZ_QXFL01000004	28942	29142	-	D2V07_RS09635		hypothetical protein
	NZ_QXFL01000004	29196	29678	-	D2V07_RS09640	*tspO*	tryptophan-rich sensory protein
	NZ_QXFL01000004	29675	30871	-	D2V07_RS09645	*bchP*	geranylgeranyl diphosphate reductase
	NZ_QXFL01000004	30868	32193	-	D2V07_RS09650	*pucC*	MFS transporter
	NZ_QXFL01000004	32190	33095	-	D2V07_RS09655	*bchG*	chlorophyll synthase ChlG
	NZ_QXFL01000004	33161	34579	-	D2V07_RS09660	*ppsR*	transcriptional regulator PpsR
	NZ_QXFL01000004	34591	35487	-	D2V07_RS09665	*btuF*	cobalamin B12-binding protein
	NZ_QXFL01000004	35698	36228	+	D2V07_RS09670	*bchF*	2-vinyl bacteriochlorophyllide hydratase
	NZ_QXFL01000004	36225	37508	+	D2V07_RS09675	*chlN*	ferredoxin:protochlorophyllide reductase (ATP-dependent) subunit N

NZ_QXFL01000004	37513	39045	+	D2V07_RS09680	*chlB*	ferredoxin:protochlorophyllide reductase (ATP-dependent) subunit B
NZ_QXFL01000004	39035	42571	+	D2V07_RS09685	*bchH*	magnesium chelatase subunit H
NZ_QXFL01000004	42585	43481	+	D2V07_RS09690	*chlL*	ferredoxin:protochlorophyllide reductase (ATP-dependent) iron-sulfur ATP-binding protein
NZ_QXFL01000004	43481	44197	+	D2V07_RS09695	*bchM*	magnesium protoporphyrin IX methyltransferase
NZ_QXFL01000004	44197	45624	+	D2V07_RS09700	*pucC*	MFS transporter
NZ_QXFL01000001	624664	625662	-	D2V07_RS03135		rhodanese-related sulfurtransferase
NZ_QXFL01000001	625906	626754	+	D2V07_RS03140	*pufL*	photosynthetic reaction center subunit L
NZ_QXFL01000001	627803	628963	+	D2V07_RS03150	*pufC*	photosynthetic reaction center cytochrome c subunit
